# Impact of Maternal Antibody on the Immunogenicity of Inactivated Polio Vaccine in Infants Immunized With Bivalent Oral Polio Vaccine: Implications for the Polio Eradication Endgame

**DOI:** 10.1093/cid/ciy649

**Published:** 2018-10-30

**Authors:** James T Gaensbauer, Chris Gast, Ananda S Bandyopadhyay, Miguel O’Ryan, Xavier Saez-Llorens, Luis Rivera, Eduardo Lopez-Medina, Mario Melgar, William C Weldon, M Steven Oberste, Ricardo Rüttimann, Ralf Clemens, Edwin J Asturias

**Affiliations:** 1 Department of Pediatrics, University of Colorado School of Medicine, Aurora; 2 Center for Global Health and Department of Epidemiology, Colorado School of Public Health, Aurora; 3 Denver Health Hospital Authority, Colorado; 4 Independent Biostatistics Consultant, Seattle, Washington; 5 Bill & Melinda Gates Foundation, Seattle, Washington; 6 Microbiology and Mycology Program and Institute of Immunology and Immunotherapy, Faculty of Medicine, University of Chile, Santiago; 7 Hospital del Nino Dr. Jose Renan Esquivel, Panama City, Panama; 8 Center for Neonatal Research, Santo Domingo, Dominican Republic; 9 Department of Pediatrics, Universidad del Valle and Centro de Estudios en Infectología Pediátrica, Cali, Colombia; 10 Hospital Roosevelt and University Francisco Marroquin School of Medicine, Guatemala City, Guatemala; 11 Centers for Disease Control and Prevention, Atlanta, Georgia; 12 Fighting Infectious Diseases in Emerging Countries (FIDEC), Miami, Florida; 13 Global Research in Infectious Diseases, Rio de Janeiro, Brazil

**Keywords:** poliovirus, maternal antibodies, IPV, bOPV, interference, schedules

## Abstract

**Background:**

Quantifying interference of maternal antibodies with immune responses to varying dose schedules of inactivated polio vaccine (IPV) is important for the polio endgame as IPV replaces oral polio vaccine (OPV).

**Methods:**

Type 2 poliovirus humoral and intestinal responses were analyzed using pre-IPV type 2 seropositivity as proxy for maternal antibodies from 2 trials in Latin America. Infants received 1 or 2 doses of IPV in sequential IPV–bivalent oral polio vaccine (bOPV) or mixed bOPV-IPV schedules.

**Results:**

Among infants vaccinated with bOPV at age 6, 10, and 14 weeks of age and IPV at 14 weeks, those with type 2 pre-IPV seropositivity had lower seroprotection rates than seronegative infants at 4 weeks (92.7% vs 83.8%; difference, 8.9% [95% confidence interval, 0.6%–19.9%]; n = 260) and 22 weeks (82.7% vs 60.4%; difference, 22.3 [12.8%–32.4%]; n = 481) post-IPV. A second IPV at age 36 weeks resulted in 100% seroprotection in both groups. Among infants vaccinated with 1 IPV at age 8 weeks followed by 2 doses of bOPV, pre-IPV type 2-seropositive infants had lower seroprotection at age 28 weeks than those who were seronegative (93.0% vs 73.9%; difference, 19.6% [95% confidence interval, 7.3%–29.4%]; n = 168). A second dose of IPV at 16 weeks achieved >97% seroprotection at age 24 or 28 weeks, regardless of pre-IPV status. Poliovirus shedding after challenge with monovalent OPV, serotype 2, was higher in pre-IPV seropositive infants given sequential IPV-bOPV. No differences were observed in the mixed bOPV-IPV schedule.

**Conclusions:**

The presence of maternal antibody is associated with lower type 2 post-IPV seroprotection rates among infants who receive a single dose of IPV. This impact persists until late in infancy and is overcome by a second IPV dose.

The global effort to eradicate polio has resulted in near-complete interruption of wild polio virus transmission [1]. After the global cessation of trivalent oral poliovirus vaccine (tOPV) use in April 2016 in favor of bivalent OPV (bOPV; types 1 and 3), vaccination with ≥1 or more doses of inactivated polio vaccine (IPV) is the only source of protection against type 2 poliovirus. Recent outbreaks of type 2 vaccine-derived poliovirus, originating primarily from previous tOPV use, highlight vulnerabilities posed to the global eradication effort owing to insufficient population immunity [1, 2]. One potential barrier to adequate individual and population immunity is the interference of maternal antibodies with infant immune responses to polio vaccination, transmitted to infants via the placenta during pregnancy or post partum in breast milk [3–5]. The first description of such interference was documented in 1958 in studies using the original IPV formulation in infants in Britain, and the finding has subsequently been demonstrated for IPV, OPV, measles, pertussis, and other vaccines [6–11]. Determining the impact of maternal antibodies on IPV immunogenicity will be important for policy makers to establish the optimal schedule to achieve adequate protection with the fewest IPV doses in the final stages of the eradication campaign.

We analyzed data from 2 recently published studies to evaluate the impact of type 2 poliovirus seropositivity before IPV administration—as a surrogate of maternally derived antibodies—on serotype 2 humoral and intestinal immunity from IPV and implications for optimal timing and number of IPV doses in low- and middle-income countries in the final stages of global polio eradication.

## METHODS

### Study Designs and Populations

Two phase IV, multicenter, randomized controlled, observer-blinded vaccine trials (IPV001 and IPV002) were conducted in 5 countries in Latin America [12, 13]. For this post hoc and unplanned analysis, we pooled a subset of infants from the original studies. The IPV001 study took place at 4 sites—Cali, Colombia; Santo Domingo, Dominican Republic; Guatemala City, Guatemala; and Panama City, Panama—and it evaluated the safety, immunogenicity, and impact on viral shedding of 1 or 2 doses of IPV in infants vaccinated with 3 doses of bOPV. From IPV001, we included infants who received bOPV at age 6, 10, and 14 weeks, and either 1 dose of IPV at 14 weeks followed by a challenge with monovalent OPV, serotype 2 (mOPV2), at 18 weeks, or 2 doses of IPV at 14 and 36 weeks, followed by an mOPV2 challenge at 40 weeks.

The IPV002 study took place in Santiago de Chile. We included in this analysis infants who received 1 dose of IPV at aged 8 weeks, bOPV at 16 and 24 weeks, and mOPV2 challenge at 28 weeks, and infants who received 2 doses of IPV at 8 and 16 weeks, 1 dose of bOPV at 24 weeks, and mOPV2 challenge at 28 weeks (for vaccine manufacturers, see Supplementary Table 1).

The primary objective of this analysis was to compare rates of type 2 poliovirus seroprotection after 1 or 2 doses of IPV, stratified by type 2 antibody status before the first IPV dose. End points related to the primary objective were seroprotection at 4 and 22 months after initial the IPV dose and 4 weeks after a second IPV dose. Secondary objectives included assessments of the impact of type 2 pre-IPV maternal antibody on post-IPV type 2 neutralizing antibody (NAb) titers, seroconversion rates at different time points, and mucosal shedding of mOPV2 after oral challenge.

### Laboratory Testing

Detailed serum and stool sample processing and laboratory assays are described in the articles reporting the primary study [12, 13]. Blood samples for type 2 polio NAb determination were obtained from all subjects in study IPV001 at age 6 weeks and at first IPV administration at 14 weeks, and then at either 18 weeks (single-dose IPV subjects) or 36 and 40 weeks (2-dose IPV subjects). In study IPV002, blood was obtained in all infants at age 8 weeks (initial IPV), 8 weeks after the final IPV dose and on the day of mOPV2 challenge (at 16 and 28 weeks in the single IPV group and 24 and 28 weeks in the 2-dose IPV group). To characterize shedding of type 2 poliovirus after mOPV2 challenge, stool samples were collected at the time of challenge and at days 7, 14, 21 and 28 after the challenge.

### Approvals and Ethics

Both studies were approved by the relevant country authorities and institutional review boards. Written informed consent was obtained from all parents or guardians [12, 13].

### Statistical Analysis

For both studies, the per-protocol population was used, which required receipt of all scheduled vaccinations. Seroprotection was defined as a NAb titer ≥8. Seroconversion in IPV001 was defined in the study protocol as either seroprotection in subjects who were previously seronegative or a minimum 4-fold increase over the expected titer level, accounting for decay of maternally derived antibody (assuming a half-life of 24 days). For seroconversion measured at week 14, the titer at week 6 was used as the baseline; for seroconversion measured at weeks 18, 36, and 40, the 14-week titer was used as baseline.

In IPV001 subjects, 4 combinations of pre-IPV serostatus were possible based on NAb measurements at age 6 and 14 weeks (seronegative/seronegative; seropositive/seropositive; seronegative/seropositive; seropositive/seronegative); median NAb, seroprotection and seroconversion rates were assessed for each group. For the primary objective, however, rates of post-IPV seroprotection were compared between subjects who were seropositive and those who were seronegative at the time of first IPV (age 14 weeks), but with the exclusion of the subset of subjects who seroconverted between 6 and 14 weeks because of potential of inadvertent environmental exposure to type 2 vaccine strain in these infants (both studies occurred before the global switch from tOPV to bOPV). In IPV002 the primary analysis was based on serostatus at the single 8-week pre-IPV time point. Intestinal immunity was assessed by extent of shedding after mOPV2 challenge using a composite quantitative shedding index end point, computed as the arithmetic mean of stool log_10_ viral titers at days 7, 14, 21, and 28 after the challenge [12, 13].

All analyses were performed with R software [14]. NAb titers and the shedding index end point were summarized with medians and bootstrap-based 2-sided 95% confidence intervals (CIs) with 10000 replicates. The Wilcoxon test was used for inference on the shedding index end point, and the log-rank method for interval-censored data was used to compare NAb titers between groups [15]. Two-sided 95% CIs for rates and differences between rates were computed via the score method, with *P* values provided by the Fisher exact method, with mid-*P* correction.

## RESULTS

**Figure 1.**
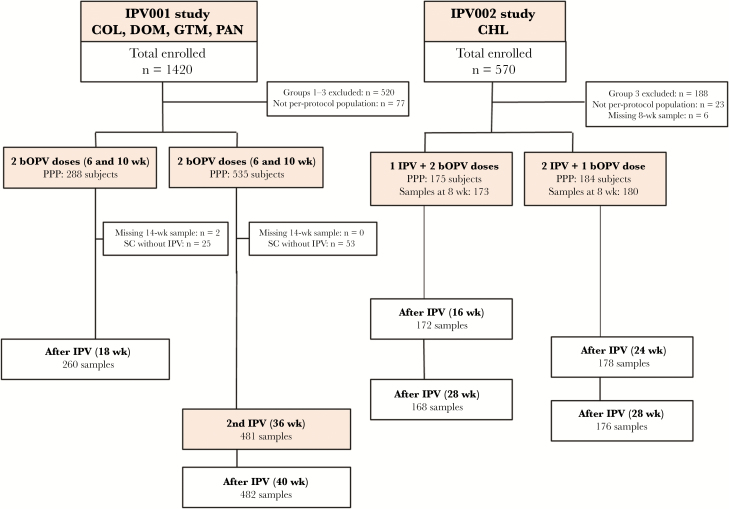
CONSORT diagram of subjects included in the inactivated polio vaccine (IPV) maternal antibody interference analysis by study. Abbreviations: bOPV, bivalent oral poliovirus; CHL, Chile; COL, Colombia; DOM, Dominican Republic; GTM, Guatemala; PAN, Panama; PPP, per protocol population; SC, seroconversion.

The per-protocol cohort was composed of 823 subjects from IPV001 and 359 from IPV002 (Figure 1). Rates of breastfeeding were high at all study sites (>94%), and no significant differences in demographic characteristics were observed between vaccination groups within the IPV001 study sites, nor between IPV001 and IPV002 (data not shown).

### Pre-IPV Serotype 2 Antibodies

From IPV001, distribution of the 4 possible combinations of pre-IPV serostatus was as follows: 330 subjects (40.2%) were seronegative at both 6 and 14 weeks of age, 223 (27.2%) were seropositive at both time points, 232 (28.3%) went from seropositive at 6 to seronegative at 14 weeks (owing to decay of maternal antibody below the lower limit of detection), and 34 (4.1%) changed from seronegative to seropositive (Table 1). The dependent variable in the primary analysis (post-IPV seroprotection) was based only on the independent variable of 14-week serostatus, excluding subjects who seroconverted between 6 and 14 weeks; this excluded group was composed of the 34 seronegative-to-seropositive subjects plus an additional 44 subjects who were seropositive at both time points but had a ≥4-fold increase in the 14-week titer over the predicted level. Thus, 743 subjects from IPV001 were included in the primary analysis: 179 (24.1%) seropositive and 564 (75.9%) seronegative at the 14-week baseline. Among IPV002 subjects, the pre-IPV type 2 seroprotection rate, measured at age 8 weeks, was 63.5%.

**Table 1.** Seroprotection, Median Titer, and Seroconversion to Type 2 Poliovirus 4 Weeks After Last IPV Dose by Serostatus at 6 and 14 Weeks of Age Among Infants in the IPV001 Study

Serostatus Categorization (From Age 6 to Age 14 wk)	Subjects, No.	Age at Measurement, wk	Seroprotection Rate (95% CI), % [No.]	Median Log Titer (95% CI)	Seroconversion Rate (95% CI), % [No.]
3 bOPV + 1 IPV dose					
SN to SN	108	18	95.4 (89.6–98.0) [103]	5.50 (5.17–6.17)	95.4 (89.6–98.0) [103]
SP to SN	84	18	89.3 (80.9–94.3) [75]	4.50 (4.00–5.17)	83.3 (73.9–89.8) [70]
SP to SP	78	18	84.6 (75.0–91.0) [66]	4.34 (3.83–4.83)	51.3 (40.4–62.1) [40]
SN to SP	15	18	86.7 (62.1–96.3) [13]	4.50 (3.17–6.50)	86.7 (62.1–96.3) [13]
3 bOPV + 1 IPV dose					
SN to SN	222	36	82.4 (76.9–86.9) [183]	4.34 (4.17–4.50)	82.4 (76.9–86.9) [183]
SP to SN	148	36	83.1 (76.3–88.3) [123]	4.50 (4.17–5.50)	83.1 (76.3–88.3) [123]
SP to SP	145	36	64.8 (56.8–72.1) [94]	3.50 (3.17–3.83)	64.8 (56.8–72.1) [94]
SN to SP	19	36	78.9 (56.7–91.5) [15]	4.50 (3.50–9.17)	78.9 (56.7–91.5) [15]
3 bOPV + 2 IPV doses					
SN to SN	222	40	100.0 (98.3–100.0) [222]	10.50 (10.50–10.50)	100.0 (98.3–100.0) [222]
SP to SN	149	40	100.0 (97.5–100.0) [149]	10.50 (10.50–10.50)	100.0 (97.5–100.0) [149]
SP to SP	145	40	100.0 (97.4–100.0) [145]	10.50 (10.50–10.50)	100.0 (97.4–100.0) [145]
SN to SP	19	40	94.7 (75.4–99.1) [18]	10.50 (10.50–10.50)	94.7 (75.4–99.1) [18]

Abbreviations: bOPV, bivalent oral polio vaccine; CI, confidence interval; IPV, inactivated polio vaccine.

### Impact of Pre-IPV Antibody Status on Serotype 2 Response to 1 Dose of IPV

**Figure 2.**
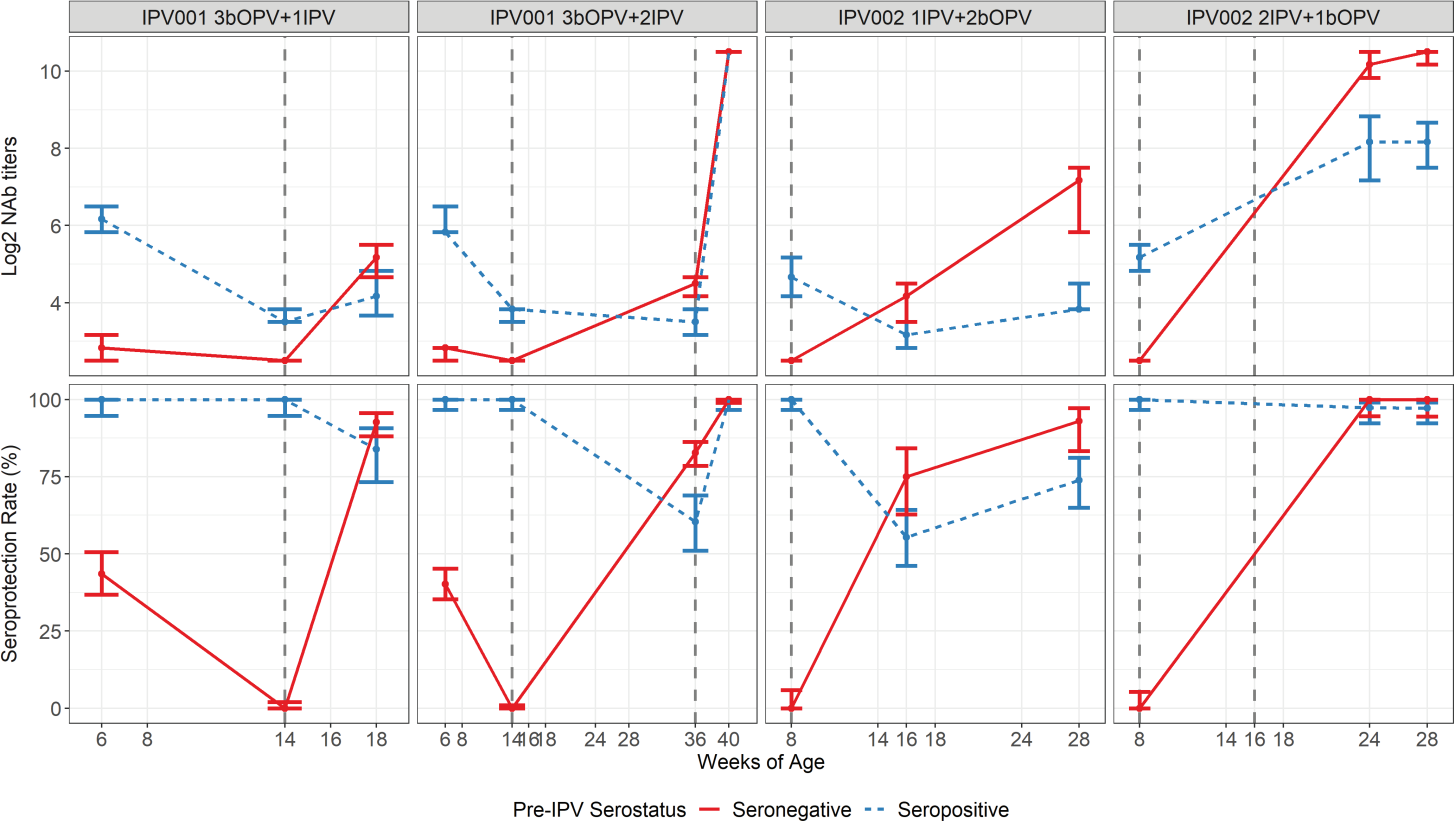
Poliovirus type 2 neutralizing antibody (NAb) titers (mean log_2_ values) and seroprotection rates (both given as estimates ± 95% confidence intervals [CIs]) according to serostatus before receipt of inactivated polio vaccine (IPV) and vaccination schedule among Latin American infants. Dashed vertical lines represent time points of IPV administration. Subjects in the IPV001 study were vaccinated with bivalent oral poliovirus (bOPV) at age 6, 10, and 14 weeks and with IPV at 14 weeks (single -IPV group) or at 14 and 36 weeks (2-dose IPV group). Subjects in the IPV002 study were vaccinated with IPV at age 8 weeks and with bOPV at 16 and 24 weeks (single-IPV group) or with IPV at age 8 and 16 weeks and bOPV at 24 weeks (2-dose IPV group).

Among infants assessed after 1 dose of IPV, there were notable differences in type 2 seroprotection rates and NAb titers between infants who were pre-IPV seronegative versus those who were pre-IPV seropositive in both studies (Tables 2, 3, and 4; Figure 2). In the IPV001 infants who received a single IPV dose, seroprotection rates measured at age 18 weeks (4 weeks after the IPV dose) were 92.7% (95% CI, 88.1%–95.6%) in the pre-IPV seronegative versus 83.8% (73.3%–90.7%) in the pre-IPV seropositive group (difference, 8.9%; .6%–19.9%; *P* = .04). Log_2_ NAb titers were 5.17 (95% CI, 4.83–5.50) in the pre-IPV seronegative versus 4.17 (3.66–4.83) the pre-IPV seropositive group (*P* = .0037). Among the infants receiving 2 IPV doses in IPV001, seroprotection rates at age 36 weeks (22 weeks after initial IPV and before the second dose) were 82.7% (95% CI,78.5%–86.2%) in the pre-IPV seronegative compared with 60.4% (51.1%–69.0%) in the pre-IPV seropositive groups.

**Table 2.** Differences in Poliovirus Type 2 Seroprotection Rates Among Latin American Infants According to Pre-IPV Serostatus and Vaccination Schedule

Study Group	Vaccine Series		
	1 IPV dose	2 IPV Doses	Full IPV + OPV Series
IPV001			
3 bOPV + 1 IPV dose^a^			
Time point	18 wk	…	…
Seroprotection, No./total; % (95% CI)			
Pre-IPV^b^ seropositive	57/68; 83.8 (73.3–90.7)	…	…
Pre-IPV seronegative	178/192; 92.7 (88.1–95.6)	…	…
Difference (95% CI), % (*P* value)	8.9 (0.6–19.9) (.04)	…	…
3 bOPV + 2 IPV doses^c^			
Time point	36 wk	40 wk	…
Seroprotection, No./total; % (95% CI)			
Pre-IPV seropositive	67/111; 60.4 (51.1–69.0)	111/111; 100.0 (96.7–100.0)	…
Pre-IPV seronegative	306/370; 82.7 (78.5–86.2)	371/371; 100.0 (99.0–100.0)	…
Difference (95% CI), % (*P* value)	22.3 (12.8–32.4) (<.001)	0.0 (−3.4 to 1.0) (>.99)	…
IPV002			
1 IPV + 2 bOPV doses^d^			
Time point	16 wk	…	28 wk
Seroprotection, No./total; % (95% CI)			
Pre-IPV seropositive	62/112; 55.4 (46.1–64.2)	…	82/111; 73.9 (65.0–81.1)
Pre-IPV seronegative	45/60; 75.0 (62.8–84.2)	…	53/57; 93.0 (83.3–97.2)
Difference (95% CI), % (*P* value)	19.6 (4.5–33.1) (.01)	…	−19.1 (−29.4 to −7.3) (.002)
2 IPV + 1 bOPV dose^e^			
Time point	…	24 wk	28 wk
Seroprotection, No./total; % (95% CI)			
Pre-IPV seropositive	…	108/111; 97.3 (92.4–99.1)	107/110; 97.3 (92.3–99.1)
Pre-IPV seronegative	…	67/67; 100.0 (94.6–100.0)	66/66; 100.0 (94.5–100.0)
Difference (95% CI), % (*P* value)	…	2.7 (2.8–7.7) (.24)	2.7 (2.9–7.7) (.24)

Abbreviations: bOPV, bivalent OPV; CI, confidence interval; IPV, inactivated polio vaccine

^a^IPV administered at age 14 weeks; bOPV at 6, 10, and 14 weeks.

^b^Pre-IPV refers to serostatus immediately before the first dose of IPV received (age 14 weeks; IPV002, age 8 weeks).

^c^IPV administered at age 14 and 36 weeks; bOPV at 6, 10, and 14 weeks.

^d^IPV administered at age 8 weeks; bOPV at 16 and 24 weeks.

^e^IPV administered at age 8 and 16 weeks; bOPV at 24 weeks.

**Table 3.** Differences in Poliovirus Type 2 Median Neutralizing Antibody Titers Among Latin American Infants According to Pre-IPV Serostatus and Vaccination Schedule: IPV001 Study Groups

Vaccine Series	Age at Measurement, wk				
	6	14	18	36	40
3 bOPV + 1 IPV dose^a^					
Titer, median (95% CI)					
Pre-IPV^b^ seropositive	6.17 (5.83–6.50)	3.50 (3.50–3.83)	4.17 (3.66–4.83)	…	…
Pre-IPV seronegative	2.83 (<2.50 to 3.17)	<2.50 (<2.50 to <2.50)	5.17 (4.83–5.50)	…	…
*P* value	…	…	.004	…	…
3 bOPV + 2 IPV doses^c^					
Titer, median (95% CI)					
Pre-IPV seropositive	5.83 (5.83–6.50)	3.83 (3.50–3.83)	…	3.50 (3.17–3.83)	>10.50 (>10.50 to >10.50)
Pre-IPV seronegative	2.83 (<2.50 to 2.83)	<2.50 (<2.50 to <2.50)	…	4.50 (4.17–4.66)	>10.50 (>10.50 to >10.50)
*P* value	…	…	…	<.001	.14

Abbreviations: bOPV, bivalent oral polio vaccine; CI, confidence interval; IPV, inactivated polio vaccine.

^a^IPV dose administered at age 14 weeks; bOPV doses at 6, 10, and 14 weeks.

^b^Pre-IPV refers to serostatus immediately before the first dose of IPV received (age 14 weeks).

^c^IPV doses administered at age 14 and 36 weeks; bOPV doses at 6, 10 and 14 weeks.

**Table 4.** Differences in Poliovirus Type 2 Median Neutralizing Antibody Titers Among Latin American Infants According to Pre-IPV Serostatus and Vaccination Schedule: IIPV002 Study Groups

Vaccine Series	Age at Measurement, wk			
	8	16	24	28
1 IPV + 2 bOPV doses^a^				
Median Titer (95% CI)				
Pre-IPV^b^ seropositive	4.66 (4.17–5.17)	3.17 (2.83–3.50)	…	3.83 (3.83–4.50)
Pre-IPV seronegative	<2.50 (<2.50 to <2.50)	4.17 (3.50–4.50)	…	7.17 (5.83–7.50)
*P* value	…	.003	…	*<*.001
2 IPV + 1 bOPV dose^c^				
Median Titer (95% CI)				
Pre-IPV seropositive	5.17 (4.83–5.50)	…	8.17 (7.17–8.83)	8.17 (7.50–8.66)
Pre-IPV seronegative	<2.50 (<2.50 to <2.50)	…	10.17 (9.83 to >10.50)	>10.50 (10.17 to >10.50)
*P* value	…	…	*<*.001	*<*.001

Abbreviations: bOPV, bivalent oral polio vaccine; CI, confidence interval; IPV, inactivated polio vaccine

^a^IPV dose administered at age 8 weeks; bOPV doses at 16 and 24 weeks.

^b^Pre-IPV refers to serostatus immediately before the first dose of IPV received (age 8 weeks).

^c^IPV doses administered at age 8 and 16 weeks; bOPV dose at 24 weeks.

A similar relationship was found among IPV002 infants in whom the seroprotection rate among pre-IPV seronegative infants at 28 weeks (after the complete regimen of 1 IPV and 2 bOPV doses and 20 weeks after IPV dosing) was 93.0% (95% CI, 83.3%–97.2%), compared with 73.9% (65.0%–81.1%) in pre-IPV seropositive infants (difference, 19.1%; 95% CI, 7.3%–29.4%; *P* = .002). The median titer was 7.17 (95% CI, 5.83–7.50) in pre-IPV seronegative infants, compared with 3.83 (3.83–4.50) in pre-IPV seropositive infants (*P* < .001).

### Impact of Pre-IPV Antibody Status on Type 2 Response to a Second IPV Dose

In the IPV001 study population, a second dose of IPV given at age 36 weeks (22 weeks after dose 1) seemed to overcome any potential interference of maternal antibody present prior to the initial IPV dose (Tables 2, 3, and 4). Among both pre-IPV seronegative and seropositive subjects, 100% were seroprotected, and median NAb titers 4 weeks after the second IPV dose were >10.50 (95% CI, >10.50 to >10.50) in both groups. Similarly, in IPV002 subjects, a second dose of IPV given 8 weeks after dose 1 (at 16 weeks) resulted in high levels of seroprotection (>97%) irrespective of pre-IPV serostatus, although higher median titers at both 24 and 28 weeks were observed among pre-IPV seronegative subjects (24 weeks, 10.17 [95% CI, 9.83 to >10.50] vs 8.17 [7.00–8.83]; 28 weeks, >10.50 [10.17 to >10.50] vs 8.17 [7.50–8.66]; both *P* < .001).

### Impact of Pre-IPV Serostatus at Age 6 and 14 Weeks on Postvaccination Seroconversion to 1 Dose of IPV

Post-IPV seroconversion was analyzed according to 6- and 14-week serostatus combinations (Table 1). Among infants who were seronegative at both 6 and 14 weeks, the seroconversion rate was 95.4% (95% CI, 89.6%–98.0%), compared with 51.3% (40.4%–62.1%) in those who were seropositive at both time points. Furthermore, a difference in maternal antibody interference was observed in the 18-week seroconversion rates between the group that was seropositive at age 6 weeks but seronegative at 14 weeks (possible maternal antibody decaying below the limit of detection between time points) and the group that was seronegative at both time points (95.4% [95% CI, 89.6%–98.0%] vs 83.3% [73.9%–89.8%]). Conversely, seroprotection rates and titers did not differ significantly between these groups.

### Impact of Baseline Antibody on Poliovirus Type 2 Intestinal Immunity

Mucosal type 2 immunity, as measured by shedding of virus after mOPV2 challenge, was compared between pre-IPV seropositive and seronegative infants (again excluding subjects who seroconverted between 6 and 14 weeks in IPV001) (Table 5). In IPV001, we noted no statistically significant impact of pre-IPV serostatus on the shedding index measured after either 1 or 2 doses of IPV. In IPV002, however, the median shedding index for both single-dose and 2-dose IPV study groups was >1 log_10_ higher (representing greater postchallenge viral shedding/weaker intestinal immunity) in pre-IPV seropositive subjects.

**Table 5.** Impact of Pre-IPV Type 2 Poliovirus Seroprotection Status on Stool Shedding Index After Oral mOPV2 Challenge, IPV001 and IPV002 Studies

Baseline Seroprotection Status^a^	Subjects, No.	Shedding Index (95% CI)	*P* Value
IPV001			
3 bOPV + 1 IPV dose			
Positive	64	2.93 (2.20–3.50)	.28
Negative	187	2.38 (1.97–2.76)	
3 bOPV + 2 IPV doses			
Positive	105	2.05 (1.51–2.66)	.81
Negative	358	2.23 (1.89–2.37)	
IPV002			
1 IPV + 2 bOPV doses			
Positive	98	3.98 (3.32–4.62)	.04
Negative	54	2.29 (1.65–3.81)	
2 IPV + 1 bOPV dose			
Positive	105	3.82 (2.85–4.62)	.002
Negative	63	2.62 (1.62–3.06)	

Abbreviations: bOPV, bivalent oral polio vaccine; CI, confidence interval; mOPV2, monovalent oral polio vaccine, serotype 2; IPV, inactivated polio vaccine.

^a^Baseline serologic status based on week 14 (IPV001) or week 8 (IPV002) serology.

## DISCUSSION

This exploratory analysis of 2 parallel studies conducted in Latin America affirms the interference of maternally derived antibodies with polio type 2 immune responses to IPV in healthy children vaccinated according to vaccine schedules endorsed in current World Health Organization guidelines [2]. The effect of maternal antibody noted after a single IPV dose was overcome by a second dose, whether administered after an 8-week interval (IPV002) and at age 4 months, or after a longer 22-week interval (IPV001) at age 9 months. Current World Health Organization guidance for countries where polio is endemic or with high risk of poliovirus importation recommends a mixed bOPV-IPV schedule and recommends that the first dose of IPV should be administered at or after 14 weeks of age to avoid the interference by maternal antibodies [2]. Our data demonstrate that even with this delayed first dose of IPV in the current schedule, vaccine responses in infants who are pre-IPV seropositive may still be impaired, leading to lower rates of seroprotection, a phenomenon noted as late as 36 weeks of life.

The biologic mechanisms of maternal antibody interference and correlation in human trials have been well characterized [16, 17]. Among 88 mother-infant pairs in the United Kingdom, post-IPV seroconversion rates increased with age of vaccination in infants receiving 2 doses of IPV starting at age 1, 6, or 10 weeks, and response was inversely correlated with the titer of maternally derived antibody at the time of the first IPV dose [6]. These observations have been subsequently confirmed in India, Puerto Rico, Pakistan, and elsewhere [7, 18, 19]. A 2017 meta-analysis demonstrated a 20%–28% decrease in post-IPV titer for every 2-fold increase in maternal antibody titer, an effect that lasted into the second year of life—consistent with observations of the durability of interference up to age 36 weeks in our study subjects [20].

In previously reported results, we noted 80% type 2 seroconversion after the single dose of IPV at age 14 weeks and nearly 98% seroprotection after the full 3 bOPV-1 IPV schedule, plus mOPV2 challenge [12]. Although this was impressive evidence of the effectiveness of a single dose of IPV in such bOPV-IPV schedules, our current analysis goes a step further in identifying a subset of individuals who may remain susceptible to type 2 poliovirus after 1 IPV dose owing to maternal antibody interference. Such findings should help inform the development of IPV schedules in the polio eradication endgame to ensure optimum immunogenicity with the fewest doses—a priority, given current IPV cost and supply issues. Two studies demonstrated good humoral [21] and intestinal [22] immune responses to IPV administered at ≥9 months of age, though all infants were previously vaccinated with tOPV. The observations in our study of 100% (IPV001) seroprotection 4 weeks after the second dose of IPV, provided at age 36 weeks, suggest that immunity induced by delayed administration of a second dose obviates the interference of maternal antibody.

Although previous data have demonstrated that delayed multiple-dose priming IPV regimens can overcome higher levels of maternal antibody, further study is warranted to determine whether the use of single-dose IPV in delayed schedules (eg, at age 9 or 12 months, concomitant with other vaccines such as measles, meningococcus A, or hepatitis A) has a similar effect [7]. An important study to determine best timing of IPV without bOPV in low- and middle-income countries is currently ongoing in Panama and the Dominican Republic (ClinicalTrials.gov NCT03239496). An alternate strategy to overcome interference of maternal antibody with a 14-week IPV may be to increase the antigen content in the IPV, thereby enhancing the 1-dose impact on serologic responses [23]. Such choices of timing of IPV dose(s) would also need to be informed by considerations regarding dropout rates for vaccines administered at later time points in routine immunization schedules, evolving epidemiology of polio, and cost and supply constraints of IPV, among other factors.

Our data demonstrate an unexpected relationship between type 2 pre-IPV seropositivity and decreased intestinal mucosal protection against subsequent mOPV2 challenge among infants in the IPV002 study vaccinated with sequential IPV-bOPV schedules starting at 8 weeks of age. This phenomenon does not seem to derive directly from the effect of maternal antibody on post-IPV serum titers, because this effect was not observed among IPV001 infants, and previous analyses have shown only weak or no correlation between prechallenge NAb titers and stool poliovirus shedding after mOPV2 challenge [24, 25]. Rather, these data suggest that the impact of maternal antibody on mucosal responses may relate to factors specific to IPV002, such as the timing and sequence of vaccination and the number of bOPV doses in the schedule. Biologically, the susceptibility in these infants likely relates to a complex interaction between maternal serum immunoglobulin G, immunoglobulin G and A in breast milk, and infant mucosal T-cell responses to bOPV [26].

Irrespective of underlying mechanism, our observations may be important for policy making in polio eradication. Polio vaccine schedules similar to that of the IPV002 study subjects have the advantage of reducing the burden of vaccine-associated paralytic poliomyelitis and are thus particularly relevant in countries where this is the predominant form of paralytic polio [2, 27]. However, because polio mucosal immunity in a population plays an important role in determining the dynamics of polio transmission [2], the interference of maternal antibody with mucosal responses after sequential IPV followed by bOPV schedules may represent an additional vulnerability to the eradication campaign.

Several limitations in our study should be acknowledged. Because this was an exploratory objective, the original studies were not powered or specifically designed for this analysis. No birth blood sample was available, resulting on our dependence on 8- or 6- and 14-week infant serum samples to represent maternal immunity. The findings from these 2 studies conducted in Latin American countries may not be generalizable to all geographic locations, particularly those where OPV has been less effective and maternal antibody levels may be higher and driven by wild or vaccine virus exposure. Passive exposure to type 2 vaccine virus could have played a role in the serologic and mucosal responses noted in the study, despite our efforts to exclude such infants from the analysis. Finally, some comparisons in this study arose from contrasts of groups across studies, which may be confounded by many other factors.

In conclusion, observations in this combined analysis of 2 large vaccine trials provide important data to inform vaccine policy for global polio eradication. The presence of type 2 maternally derived antibodies was associated lower rates of seroprotection in infants who received a single dose of IPV, an effect that was still prominent at 36 weeks of life. This interference can be overcome by adding a second IPV dose. Future research to further describe this phenomenon, and establish whether the pattern of such interference is also observed with newer and future formulations of IPV currently under development, will be important [28, 29].

## Supplementary Data

Supplementary materials are available at *Clinical Infectious Diseases* online. Consisting of data provided by the authors to benefit the reader, the posted materials are not copyedited and are the sole responsibility of the authors, so questions or comments should be addressed to the corresponding author.

## Supplementary Material

Supplemental_Table

## Notes

***Author contributions.*** All authors contributed to the study design, data interpretation and writing of the manuscript. All other authors share final responsibility for the decision to submit for publication.

***Acknowledgments.*** We thank Joel Ward and Sue Ann Costa-Clemens for their contributions to the design and analysis of these studies; Ciro de Quadros, Maria Teresa Valenzuela, Fernando Lolas, Claudio Struchiner, and Carolina Danovaro for study oversight as the Data Safety Monitoring Board; Steve Self and Bhavesh Borate for support in data analysis; Daniel Stamboulian and the Fighting Infectious Diseases in Emerging Countries team for its coordination and support to the study; Bilthoven Biologicals, GlaxoSmithKline, and Sanofi Pasteur for kindly donating vaccines to the study. Thanks also to laboratory staff at the Centers for Disease Control and Prevention, in Atlanta Georgia: Deborah Moore, Yiting Zhang, Sharla McDonald, William Hendley, Larin McDuffie, and Mario Nicolas. Other investigators who participated in the IPV001 study include Pio Lopez, Viviana Marquez, and Elizabeth Toro, Centro de Estudios en Infectología Pediátrica, Cali, Colombia; Ingrid L. Contreras-Roldan and Diva M. Calvimontes, University del Valle Guatemala, Guatemala; Jose Jimeno, Ana C. Villarreal, and John J. Solano-Buitrago from Cevaxin, Panama City, Panama; Lourdes Peña and Herny Julian from the Center for Neonatal Research, Santo Domingo, Dominican Republic; and Rodolfo Villena from the University of Chile, Santiago, Chile.

***Financial support.*** The IPV001 and IPV002 studies were funded by the Bill & Melinda Gates Foundation.

***Supplement sponsorship.*** This article appears as part of the supplement “Polio endgame and beyond: Vaccine choices, transmission dynamics, and surveillance implications,” sponsored by The Bill & Melinda Gates Foundation.

***Potential conflicts of interest.*** A. S. B. is a full-time employee at Bill & Melinda Gates Foundation (BMGF). E. L. M. reports grants from Centro de Estudios en Infectología Pediátrica and from BMGF, during the conduct of the study. M. M. reports personal fees from the University of Colorado, during the conduct of the study, and from Sanofi Pasteur, outside the submitted work. E. J. A .reports grants from BMGF, during the conduct of the study, and grants from GlaxoSmithKline and Takeda Vaccines, outside the submitted work. All other authors report no potential conflicts. All authors have submitted the ICMJE Form for Disclosure of Potential Conflicts of Interest. Conflicts that the editors consider relevant to the content of the manuscript have been disclosed.
