# Supplementary material for: Impact of Maternal Antibody on the Immunogenicity of Inactivated Polio Vaccine in Infants Immunized With Bivalent Oral Polio Vaccine: Implications for the Polio Eradication Endgame
Source: Clin Infect Dis. 2018 Oct 30;67(Suppl 1):S57–65. doi: 10.1093/cid/ciy649 (PMC6206111; doi:10.1093/cid/ciy649)
Supplement: Supplemental_Table [file ciy649_suppl_supplemental_table.docx]

Supplemental Table: Manufacturers of polio vaccine used in the IPV001 and IPV002 Latin American studies

| bOPV | Sanofi Pasteur, Lyon, France |
| --- | --- |
| IPV | Sanofi Pasteur, Marcy L’Etoile, France |
|  | GlaxoSmithKline, Wavre, Belgium |
|  | Bilthoven Biologicals, Bilthoven, the Netherlands |
| mOPV2 | Polio Sabin Mono Two (oral), GlaxoSmithKline, Rixensart, Belgium |
